# Using Massive Parallel Sequencing for the Development, Validation, and Application of Population Genetics Markers in the Invasive Bivalve Zebra Mussel (*Dreissena polymorpha*)

**DOI:** 10.1371/journal.pone.0120732

**Published:** 2015-03-17

**Authors:** Luis Peñarrubia, Nuria Sanz, Carles Pla, Oriol Vidal, Jordi Viñas

**Affiliations:** Laboratori d’Ictiologia Genètica, Departament de Biologia, Universitat de Girona, Campus Montilivi, Girona, Spain; University of Innsbruck, AUSTRIA

## Abstract

The zebra mussel (*Dreissena polymorpha*, Pallas, 1771) is one of the most invasive species of freshwater bivalves, due to a combination of biological and anthropogenic factors. Once this species has been introduced to a new area, individuals form dense aggregations that are very difficult to remove, leading to many adverse socioeconomic and ecological consequences. In this study, we identified, tested, and validated a new set of polymorphic microsatellite loci (also known as SSRs, Single Sequence Repeats) using a Massive Parallel Sequencing (MPS) platform. After several pruning steps, 93 SSRs could potentially be amplified. Out of these SSRs, 14 were polymorphic, producing a polymorphic yield of 15.05%. These 14 polymorphic microsatellites were fully validated in a first approximation of the genetic population structure of *D*. *polymorpha* in the Iberian Peninsula. Based on this polymorphic yield, we propose a criterion for establishing the number of SSRs that require validation in similar species, depending on the final use of the markers. These results could be used to optimize MPS approaches in the development of microsatellites as genetic markers, which would reduce the cost of this process.

## Introduction

The zebra mussel (*Dreissena polymorpha*, Pallas, 1771) is a successful invasive bivalve that is native to the brackish and fresh waters that drain into seas of the Ponto-Caspian region (Black, Caspian, and Azov Seas) [1]. This species is now considered one of the World's 100 most invasive species according to the International Union for Conservation of Nature—Invasive Species Specialist Group (IUCN—ISSG) [2]. *Dreissena polymorpha* has several biological attributes that facilitate the success of its invasions, including rapid growth with early sexual maturity, dispersal by larvae, unspecific food preference, and gregarious behavior [3]. The invasive behavior of this species is further enhanced by several transport-related anthropogenic factors, primarily the ballast water of boats that move through inland waterways [4].

The freshwater invasion of *D*. *polymorpha* is of public interest because of the associated major economic and ecological damage caused by this species. Once individuals are introduced to a new area, high-density aggregations [3] have many adverse socioeconomic and ecological consequences including habitat destruction, the loss of species diversity, and the extinction of native species [5]. The socio-economic effects of dense colonies of *D*. *polymorpha* include the inability usage of freshwater superficies for recreational purposes and the blockage of artificial water-conducts, among others. The latter creates serious working problems to industries and water supply systems, as well as to the watering and refrigeration systems of hydroelectric, thermic, and nuclear structures [6]. Understanding the population genetic structures in invasive species have revealed their usefulness for inferring source regions, routes of invasion and possible threatened locations by phylogeographic analysis. In recent years, molecular markers have been increasingly used on Dreissenid species in studies of taxonomy, phylogeny, genetic diversity, and phylogeography [7]. The mitochondrial gene of the Cytochrome Oxidase subunit I (COI) and nuclear Amplified Fragment Length Polymorphism (AFLP) markers have been used to examine the evolutionary history of *D*. *polymorpha* species in Europe [8–9], revealing highly structured populations. Nuclear allozyme markers have been used to analyze the North American invasion indicating that its presence was consequence of accumulative invasion events from Europe [10–11].

Microsatellites [12], also known as Simple Sequence Repeats (SSRs) or Short Tandem Repeats (STRs), are one of the most informative types of molecular markers due to their high variability, consequence of their high mutation rates, and power to resolve population structure, even among closely related populations [13]. Other advantages of microsatellite markers are that they are abundant in the genome, co-dominant and easy to detect by PCR [14–15]. Consequently, microsatellite loci have been the most used genetic markers for population genetic analysis, and they are used in a diverse range of applications, such as parentage analyses, genetic mapping, and conservation genetics [16]. These markers have also proven to be useful for the genetic characterization of invasive species [17–18], where genetic diversity is reduced [19], as well as to infer the sources and pathways of the introduced populations in aquatic ecosystems [20–21]. However, despite the critical situation caused by the expansion of *D*. *polymorpha* across Europe and North America, only a few microsatellites have been described for this species [22–24] and their analyses have corroborated the results obtained by mitochondrial and allozyme markers [20, 25].

For most non-model species, few or none microsatellite are described, and they must be isolated *de novo*. One of the causes of having limited number of microsatellite loci is consequence of the high cost and labor-intensive approaches required in traditional methodologies of microsatellites isolation [26–27], but are being overcome by new sequencing technologies. For instance, large databases of genomic sequences have been obtained by Massive Parallel Sequencing (MPS) methods (also known as Next-Generation Sequencing or NGS), which could be screened using bioinformatics tools to detect large sets of microsatellite markers [28], even in invasive mollusk species [29].

All of the microsatellites identified in *D*. *polymorpha* employed traditional hybridization probes. The implementation of new microsatellites by MPS methods may provide new markers for assessing the dispersion capacity and invasion routes of *D*. *polymorpha*, which would help with the establishment of management strategies to control the invasive potential of this species [30]. Thus, the main objective of this study was to identify and validate new polymorphic microsatellite loci using MPS platforms to increase the number of molecular markers for *D*. *polymorpha*. This process involved establishing procedures to set up and implement a protocol for the identification and validation of new markers for application in successive non-model species and organisms. The second objective was to determine the number of microsatellite markers needed for several genetic applications. The newly developed markers are expected to be used in subsequent studies focusing on the population genetic structure of *D*. *polymorpha*, to determine its invasion history and dispersal routes through Europe. This information could potentially help predict possible new areas of invasion, which would allow the implementation of preventative management to prevent accidental introduction.

## Methods

### Phase A: MPS Data Processing

#### DNA Extraction and Sample Selection

**Fig 1:**
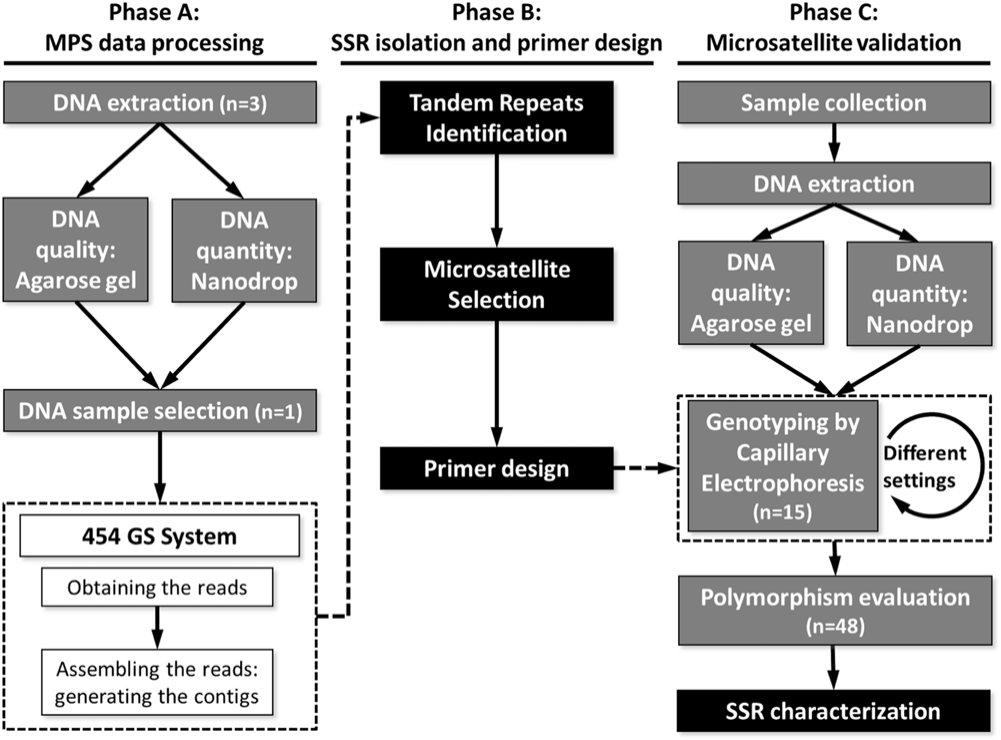
Experimental design and protocol pipeline. Gray steps: Laboratory protocols. White steps: 454 GS System. Black steps: Bioinformatic analysis.

Total DNA was isolated separately from three *D*. *polymorpha* individuals by two phenol-chloroform and ethanol precipitations, as described in [31]. RNase treatment was performed between the two DNA precipitations by adding 2 μL of RNase (10 μL/ml) in each DNA tube, and implementing an incubation step of 30 min at 37°C. DNA quality was analyzed by 0.6% Agarose Gel Electrophoresis, and DNA quantity was evaluated using the Nanodrop spectrophotometer ND-1000 (Thermo Fischer Scientific, Inc. Waltham, Massachusetts, U. S. A.) (Fig. 1). Finally, the best of the three DNA extractions was sent for the massive DNA sequencing at CRAG (Centre de Recerca en Agrigenòmica, Barcelona, Spain).

#### Sequencing by the 454 GS System

DNA was sequenced using a 454 GS FLX platform using 1/8 of a plate. Reads were assembled using GS DE NOVO ASSEMBLER software, version 2.5.3. (Fritz Hoffmann-La Roche Ltd. Basel, Switzerland) (Fig. 1). Two strategies were used to compare different yield when recovering reads into the contigs. First, we used the default align and assemble parameters: minimum read length = 20 base pairs (bp); seed step = 12 bp; seed length = 16 bp; seed count = 1; minimum match length = 40 bp; overlap minimum match identity = 90%; alignment identity score = 2; alignment difference score = 3; and minimum output sequence size = 100 bp. Second, we ran an assembly to optimize the number of assembled reads into the contigs. We used the same assembled parameters and included the options of “Large or Complex Genome” to specify that the species was eukaryotic and “Heterozygotic Mode,” due to the diploid nature of *D*. *polymorpha*.

### Phase B: SSR Isolation and Primer Design

#### Identification and Microsatellite Selection of Tandem Repeats

We used TANDEM REPEATS FINDER (TRF) software version 4.04 [32] to identify simple tandem repeats (Fig. 1). TRF target SSR sequences were aligned to a predefined library of consensus tandem repeats sequences. We selected alignments of 30 nucleotides, with values of match = 2, mismatch = 7, and indels = 7, and a maximum period size of five nucleotides for each microsatellite. This final parameter excludes hexanucleotide SSRs. Homopolymeric repeats were discarded, due to the extremely high number of sequencing errors made within homopolymeric runs using the 454 GS FLX platform [33].

Tandem repeat structures identified by TRF were pruned to obtain the Potential Amplifiable Loci (PAL), after removing structures composed of less than five tandem repetitions and regions with less than 30 base pairs in each of the flanking sequences and with positive primer annealing positions. In addition, we considered two neighboring SSRs separated by less than 100 nucleotides as the same locus (a compound microsatellite).

#### Primer Design

PCR primers were designed (Fig. 1) using PRIMER3 software, version 0.4.0. [34]. BLASTN [35] analysis was run to discount possible homologies that could produce inespecifities in the PCR. Predicted amplicons spanned three size ranges (100–150, 200–300, and 350–450 base pairs) to facilitate possible implementation in future multiplex PCR without overlapping allele sizes. All forward PCR primers contained a sequence of 19 additional nucleotides (CACGACGTTGTAAAACGAC) at each 5ʹ-end. A labelled primer consisting of the same sequence with a 6-FAM fluorochrome was included in each genotyping PCR, as described in Schuelke *et al*. [36].

### Phase C: Microsatellite Validation

#### Sample Collection and DNA Extraction

Forty-eight *D*. *polymorpha* individuals were collected from six representative locations where they had been introduced in north-eastern Spain (S1 Table). Samples were collected and manipulated under permits provided by the Catalan Water Agency of the Agriculture, Fisheries, Food and Environment Department of the Autonomous Community of Catalonia and the Hydrographic Confederation of the Ebro River of the Agriculture, Food and Environment Ministry of Spain. All work was performed in compliance with and approved by the Ethics Committee of the University of Girona and met the requirements stated by the Spanish (RD53/2013) and Catalonian (D214/1997) laws of animal care and experimentation. The shell was removed, and the whole muscle bodies were preserved in 96% ethanol until processing. DNA extraction was performed using the Real Pure DNA Extraction Kit (Durvitz, Valencia, Spain). Quality was checked using 0.6% Agarose Gel Electrophoresis, and DNA quantity was measured with a Nanodrop spectrophotometer ND-1000 (Thermo Fischer Scientific, Inc. Waltham, Massachusetts, USA) (Fig. 1).

#### Genotyping and Polymorphism Evaluation

The PCR mix was prepared in a final volume of 20 μL, containing 1X Buffer (BIOLINE), 1.5 μM MgCl_2_, 0.8 mM dNTPs, 5x10^-3^ μM primer Forward, 0.2 μM primer Reverse, 0.2 μM 6-FAM dye-probe, and 2.5x10^-2^ u/μL Taq polymerase (BIOLINE). DNA samples (25–100 ng) were added to each PCR mix. The thermal cycles consisted of an initial denaturing step of 3 min at 94°C, followed by 35 cycles of denaturing at 94°C for 30 s, annealing at 50°C or 60°C for 90 s, and extension at 72°C for 90 s, and a final extension period of 10 min at 72°C. Negative controls were included in all PCR runs to confirm that there was no cross-contamination. PCR products were read in a 3130 Genetic Analyzer (Applied Biosystems).

Validation analysis of PALs was conducted in two steps (Fig. 1). First, PCR screening of the putative SSRs was completed to check the specificity of the primers in 15 *D*. *polymorpha* individuals from four different locations separated in range of 167 and 562 km. If these initial PCR conditions failed or unspecific results were obtained, a second attempt was made using the same samples, but increasing the annealing temperature to 60°C. Successfully validated loci were genotyped in 48 *D*. *polymorpha* individuals to assess polymorphism and allele richness.

#### SSR Characterization

We evaluated the presence of null alleles in each locus using MICRO-CHECKER software, version 2.2.3 [37]. The observed (*H*_*o*_) and expected (*H*_*s*_) heterozygosities were assessed for each new polymorphic locus for conformance to Hardy-Weinberg and Linkage disequilibrium expectations by the exact test implemented in GENEPOP 4.0 software [38]. We calculated the Polymorphism Information Content (*PIC*) index to estimate the information values of each microsatellite marker [39]. Microsatellite sequences were added to the GenBank Database with accession numbers [GenBank: JQ812984-JQ812997].

We also tested the 14 new polymorphic microsatellites in the preliminary characterization of the genetic structure of *D*. *polymorpha* in the Iberian Peninsula. We analyzed 48 *D*. *polymorpha* individuals from six representative locations where they are present in north-eastern Spain (S1 Table). The Hardy-Weinberg equilibrium (*HWE*) of genotypic distributions was measured at each site by the exact probability test [40] using GENEPOP software version 4 [38]. Benjamini & Yekutieli [41] False Discovery Rate (FDR) was applied to each test to adjust the significance levels for multiple simultaneous comparisons. Genetic diversity within each study site was estimated from direct counts as the number of alleles per locus (*A*), the estimated expected heterozygosity (*H*_*s*_), and allelic richness (*A*_*r*_) from allele frequencies using FSTAT 2.9.3 [42]. In addition, pairwise population differentiation (*F*_*ST*_) [43] and significance values were calculated using FSTAT software. The number of genetically homogeneous groups (*K*) was estimated among all sampled collections using the Bayesian Markov Chain Monte Carlo approaching method of STRUCTURE software version 2.3.2 [44]. Runs for each possible *K* (1 to 6) were repeated five times. A burn-in period of 20,000 steps, followed by 100,000 Monte Carlo replicates, was simulated with the model of independent allele frequencies. The optimal *K* value was selected following the recommendations of Pritchard et al. [44], according to the posterior probability of the data. Finally, patterns of gene diversity distribution among *D*. *polymorpha* collections provided by the genetic results were estimated by Analyses of Molecular Variance (AMOVA) conducted in ARLEQUIN 3.5 [45]. In the AMOVA model, all six locations were grouped into two hydrographic basins (i. e., the Ebro River and Llobregat River). We tested whether genetic diversity was partitioned into three levels: within locations, among locations within river basins, and among river basins.

## Results

### Phase A: MPS Data Processing

#### 454 GS System Sequencing Results

The 454 GS FLX run generated 110,593 reads (read range size = 24–691 bp, mean read size = 331 bp), with a total of 36,554,817 bases (Table 1). GS DE NOVO ASSEMBLER software generated two assembled outputs based on the selected parameters (see Methods section). In the first output using the default parameters, 31,330 reads (28.33% of total) were assembled into 2,326 contigs (contig range size = 100–8,697 bp, mean contig size = 457 bp, N50 = 825 bp) and 70,208 singletons (Table 1). In comparison, the second output using modified parameters assembled 35,374 reads (31.99% of total) into 3,885 contigs (contig range size = 100–8,717 bp, mean contig size = 421 bp, N50 = 860 bp) and 68,463 singletons (Table 1). Since one of the main objectives of this study was to find as many *SSR* structures as possible, we selected the second assembly with the modified parameters “Large or Complex Genome” and “Heterozygotic Mode,” because it generated a higher N50 value with a greater number of long contigs of the same sequence quality.

**Table 1:** Summary of MPS results and of polymorphic microsatellites identification.

		Output description	Default assembly	Modified assembly
Phase A	Sequencing step	Reads number	110,593	
		Largest read size (bp)	691	
		Shorter read size (bp)	24	
		Reads mean size (bp)	331	
		Sequenced bases (Kb)	36,555	
	Assembling step	Aligned reads (of the total right reads)	31,330 (28.33%)	35,374 (31.99%)
		Aligned bases (Kb) (of the sequenced bases)	7,620 (20.84%)	9,030 (24.71%)
		Assembled reads	18,758	20,570
		Contigs number	2,326	3,885
		Bases number into contigs (kb)	1,063	1,635
		Largest contig size (bp)	8,697	8,717
		Shorter contig size (bp)	100	100
		Contig mean size (bp)	457	421
		Contig N50 value (bp)	825	860
		Singletons number	70,208	68,463
Phase B		Initial *SSR* identified	-	288
		Potential Amplifiable Loci (% initial SSR)	-	93 (32.29%)
Phase C		Positive PCR amplification (% initial SSR)	-	81 (28.13%)
		Polymorphic markers (% SSR)	-	14 (4.86%)

Phase A: MPS data processing; Phase B: SSR isolation and primer design; Phase C: Microsatellite validation

### Phase B: SSR Isolation and Primer Design

#### Identification, Microsatellite Selection, and Primer Design of Tandem Repeats

*TRF* software identified a total of 299 single SSR structures. After removing homopolymers, 288 SSRs were identified with an overall estimated density of 0.18 SSRs/Kb. The SSRs were distributed in 6.60%, 37.85%, 39.58%, and 15.97% of di-, tri-, tetra-, and pentanucleotides respectively (S2 Table). Of these SSRs, 93 (32.29% of the 288 initial SSRs identified that had a density of 0.06 SSRs/Kb) were identified as Potential Amplifiable Loci (PAL), after removing sequences with less than 5 units of repetition or with less than 30 nucleotides at each end of the tandem repeat, assuming that repeat structures separated by less than 100 base pairs were the same locus, and with positive designed PCR primers (Table 1 and S3 Table).

### Phase C: Microsatellite Validation

#### Genotyping and Polymorphism Evaluation

All 93 PALs were tested for PCR amplification in 15 representative *D*. *polymorpha* individuals out of the 48 individuals collected from six locations in the North-Eastern Iberian Peninsula (S1 Table). Twelve PALs failed to produce PCR products. The remaining 81 loci, which generated positive PCR amplification with a product within the expected size range, were genotyped in the set of 48 mussel samples. These loci represented 28.13% of the 288 initially identified SSRs (Table 1). Fourteen of these 81 loci showed polymorphism (Table 2). Blast analysis of these new 14 polymorphic microsatellites resulted in no matches with previously published microsatellite markers in *D*. *polymorpha*.

**Table 2:** Polymorphic microsatellites description.

Locus	Repeat motif	*S*	Primer Sequences 5’ → 3’	*Ta*	*A*	*H*_*o*_ */H*_*s*_	*PIC*	*NA*	*LD*	GenBank Accession
*Dp1*	[ATA]_21_	279–299	*F*: GGATTTTTCTCCCGTGGAAT	60	5	0.83 / 0.81	0.76	No	No	JQ812984
			*R*: CGGTAGCGTTCTCTTCACAA							
*Dp2*	[TGA]_17_	410–440	*F*: GCTACCGGAGCTCAACCTAA	60	9	0.20 / 0.66	0.62	Yes	No	JQ812985
			*R*: ACGTCGAACCCTGTCAAAAA							
*Dp7*	[TAA]_11_ 4 [ATT]_5_ 48 [TAT]_11_ 71 [ATA]_14_	411–416	*F*: GGAATACCGGGTGCTGTAGA	50	2	0.28 / 0.49	0.37	No	No	JQ812986
			*R*: GACGTGCGTCACAATAGGTG							
*Dp30*	[TTG]_7_ 57 [GTT]_6_	214–234	*F*: GCGTTGGTGTTGTGTACGTC	60	6	0.58 / 0.67	0.62	No	No	JQ812987
			*R*: CTGAGCATCTCACCGTCAAA							
*Dp31*	[ATT]_13_	231–306	*F*: CGAGTTTCTTGCACGTTTCA	50	13	0.79 / 0.86	0.83	No	No	JQ812988
			*R*: TGTTATTTTAAGAAGGCCACATTG							
*Dp39*	[GGCG]_11_	385–393	*F*: GACGTCATGGTTCTGAATGG	50	3	0.56 / 0.66	0.58	No	No	JQ812989
			*R*: CCGGACAAGCTCATTTATGG							
*Dp42*	[GTTG]_9_	243–256	*F*: TCGCTTAACCTGACCAGTGA	50	3	0.60 / 0.59	0.52	No	No	JQ812990
			*R*: CCAAATATCAAGTTGCCTATCTTCA							
*Dp43*	[TTA]_10_	224–259	*F*: TTGCTCATGATGAAATATGATGT	50	5	0.34 / 0.38	0.35	Yes	No	JQ812991
			*R*: ATGCGTTTCACTTTGGCATC							
*Dp44*	[GACC]_8_	139–153	*F*: CCCCAAGCGTCTTGAGTATC	50	5	0.60 / 0.67	0.60	No	No	JQ812992
			*R*: TCCTGCCAAGCATGTATGAG							
*Dp68*	[TGTTC]_5_	291–297	*F*: TGCTACACACCGTATTTGCTG	50	3	0.23 / 0.21	0.19	No	No	JQ812993
			*R*: ACACGTGGATGGTGTGAAGA							
*Dp72*	[GGTA]_8_	378–382	*F*: TGCACACACATCTTGACCTG	50	2	0.31 / 0.41	0.32	Yes	No	JQ812994
			*R*: GCTGAAGGCACAACATTTGA							
*Dp74*	[CGTC]_9_	332–360	*F*: ATCCCCTCAAGACGTTTCCT	60	3	0.57 / 0.58	0.49	No	No	JQ812995
			*R*: ACCATACCGGTGGCATAAAA							
*Dp86*	[CGTC]_5_	306–315	*F*: GCAAAGGGAGAAAACTGCAC	50	4	0.73 / 0.71	0.65	No	No	JQ812996
			*R*: CACTGTCACCGTCGCACATA							
*Dp89*	[CGTC]_8_	260–284	*F*: TTTTCACACAGCAGCCAAAG	50	4	0.56 / 0.53	0.42	No	No	JQ812997
			*R*: TGAGAAATAGCCCGGACAAA							

Repeat motif, size range in base pairs (*S*), forward (*F*) and reverse (*R*) (5′– 3′) sequences, annealing temperatures in°C (*Ta*), number of alleles (*A*), Observed (*H*_*o*_) and Expected (*H*_*s*_) heterozygosities, *PIC* index, presence of null alleles (*NA*), linkage disequilibrium (*LD*) and GenBank accession number for the 14 polymorphic microsatellites loci tested on 8 individuals for each of the 6 populations (*n* = 48) in *D*. *polymorpha*.

#### SSR Characterization

MICRO-CHECKER software detected null alleles or scoring errors in three (*Dp2*, *Dp43*, and *Dp72*) of the 14 microsatellite loci (Table 2). The *Dp2* locus presented null alleles at three of the locations, whereas *Dp43* and *Dp72* presented null alleles at just one location. The number of alleles detected at each locus ranged from two (*Dp7* and *Dp72*) to 13 (*Dp31*). Estimates of gene diversity and the *PIC* index varied among loci. The lowest value of expected heterozygosity was obtained for *Dp2* (*H*_*s*_ = 0.20), while the highest value was obtained for *Dp1* (*H*_*s*_ = 0.83). The lowest *PIC* index was in *Dp68* (*PIC* = 0.19), while the highest was in *Dp31* (*PIC* = 0.83) (Table 2). Only two markers (*Dp1* and *Dp2*) presented significant deviations from Hardy-Weinberg equilibrium at the 5% significance level, and all loci appeared to be unlinked, according to the lack of gametic disequilibria in each location (Table 2).

These validated microsatellites were used for the preliminary analysis of the genetic population structure of *D*. *polymorpha* in the Iberian Peninsula. All 48 individuals collected from the six locations were used, with a sample size of eight individuals from each sampling site (five locations from the Ebro River and one from the Llobregat River; S1 Table). All 14 microsatellite loci were polymorphic at all six locations. Analyses of allele frequencies (S4 Table) presented deviations from *HWE* in only one location at Ebro River. After adjusting for differences in sample size, permutation tests demonstrated lower average allelic richness (*A*_*r*_) and genetic diversity (*H*_s_) in the Llobregat River (*A*_*r*_ = 2.06; *H*_*s*_ = 0.50) compared to the Ebro River basin (*A*_*r*_ = 2.14–2.33; *H*_*s*_ = 0.53–0.61). The lowest number of total alleles (*A*) across all 14 loci also corresponded to the Llobregat River (*A* = 43) in comparison to the Ebro River (*A* = 46–50) (S1 Table).

Estimated average genetic differentiation (*F*_*ST*_) among locations was *F*_*ST*_ = 0.044. None of the pairwise *F*_*ST*_ values were significant after FDR correction (S5 Table). The Bayesian analyses of STRUCTURE showed an *ln P* value that was lower for *K* = 1 compared to for *K* = 2 and *K* = 3 (*K* = 1, mean *ln P* = -1429.80; *K* = 2, mean *ln P* = -1473.96; *K* = 3, mean *ln P* = -1582.28). Following the recommendations of Pritchard et al. [38], these results distinguished only one genetically homogeneous group of populations for all analyzed locations (*K* = 1). Finally, the AMOVA test revealed similar genetic variance between the tested river basins (3.20%) and among locations within river basins (3.89%). Furthermore, genetic variance was not significant (*P* = 0.062) in the comparison between the two river basins.

## Discussion

### New Microsatellites in *D*. *polymorpha*

This study presents a set of new polymorphic microsatellites in *D*. *polymorpha* (Table 2), which, together with previously described microsatellites [22–24], represent a useful tool for investigating genetic variation within and among populations of this species.

This study is the first to use MPS platforms to discover new SSRs in *D*. *polymorpha*. Interestingly, our approach yielded a higher number of initially identified SSR structures (288 SSRs in this case) compared to the average number of SSR structures identified using hybridization methodologies (mean = 47 SSRs, *SD* = 18.52) (Table 3). However, the polymorphic yield (ratio between the number of polymorphic microsatellites divided by the number of PCR validated loci) of hybridization techniques has an average of 64.58% of final polymorphic markers, which is substantially higher than the 15.05% obtained in our analysis using MPS (Table 3). Yet, the final number of polymorphic microsatellites obtained by MPS is greater than the expected total number of microsatellites obtained by hybridization techniques (Table 3).

**Table 3:** Microsatellites described in *Dreissena polymorpha*.

		Initial SSR identified	PAL loci	Polymorphic microsatellites	Polymorphic yield	Reference
			(% of initial SSR)			
Traditional techniques		65	16 (24.62%)	5	31.25%	[18]
		48	8 (16.67%)	8	100.00%	[19]
		28	8 (28.57%)	5	62.50%	[20]
	Mean	47	10.67 (23.40%)	6	64.58%	
	*SD*	18.52	4.62	1.73	34.42	
This study		288	93 (32.29%)	14	15.05%	This study

Initial SSR identified, PAL loci, Polymorphic microsatellites, and Polymorphic yield in all studies describing microsatellite loci in *D*. *polymorpha*. *SD*: Standard deviation. PAL: Potential Amplifiable Loci. Polymorphic yield is the ratio between the numbers of polymorphic microsatellites divided by the number of PCR validated loci.

Microsatellite isolation and validation in bivalve species is challenging for several reasons. For instance, we found about180 microsatellites per Mb in *D*. *polymorpha*. This value is situated in the lower range of 50–1,500 microsatellites/Mb expected in eukaryotes [46]. This phenomenon is probably related to the large genome of bivalves, particularly for *D*. *polymorpha* (1.70 ± 0.03 pg, about 1.6 Gb) [47–48], combined with the suspected low frequency of microsatellites in the bivalve genome [26, 49]. In addition, PCR design for microsatellite amplification is not always efficient because of the high number of repetitive elements in the flanking regions of mollusks [50]. On average, invertebrates require over twice as many sequences to obtain the same number of useable loci compared to other taxa, like plants and vertebrates [28].

In the current study, we obtained a slightly lower values for the average number alleles per locus (*A* = 4.79) and mean gene diversity (*H*_*s*_ = 0.56) compared to the value obtained by previous studies analyzing the population genetic structure of *D*. *polymorpha* (*A* = 12.70, H_s_ = 0.84, 5 *SSRs*, [25]; *A* = 35.09, *H*_*s*_ = 0.61, 11 microsatellites; [20]). The present study focused on validating and obtaining a first approximation of the usefulness of the developed markers in a very restricted number of *D*. *polymorpha* individuals (*n* = 48) compared to the sample sizes used by previous studies (*n* = 309 [25] and *n* = 386 [20]). This difference may partly explain the lower genetic diversity detected here. However, the overall genetic diversity obtained here was adequate for use in further genetic studies.

### Microsatellites in Population Genetics Analyses and Other Applications

Microsatellite markers are the most used genetic markers for population genetic analysis, due to their high variability and power to resolve population structure, even among closely related populations [13]. To optimize the laboratory work in population genetic analyses, it is crucial to determine the minimum number of microsatellite markers used. Then, two issues must be considered: 1) how many microsatellites are needed to achieve a specific objective; and 2) how much effort is needed to develop the desired number of polymorphic microsatellites using MPS platforms. Chistiakov *et al*. [16] classified six main applications of microsatellites (Table 4): 1) genetic mapping; 2) individual DNA identification and parentage assignment; 3) phylogeny, population, and conservation genetics; 4) molecular epidemiology and pathology; 5) quantitative trait loci mapping; and 6) marker-assisted selection. However, the authors did not infer the average number of microsatellites required for each specific objective. To gather this information, we reviewed all 104 studies included in the analysis by Chistiakov *et al*. [16] (S6 Table). We used the median value to avoid bias caused by extreme values present in some of the applications. As expected, genetic mapping requires the highest number of microsatellites (median = 209), whereas a median of just four microsatellites is required for marker-assisted selection studies. In molecular epidemiology and pathology, only one microsatellite is necessary when the diagnostic marker associated to a metabolic disorder is known, while several (hundred) markers are used to search for new diagnostic markers. For population genetic studies, which are the target application for *D*. *polymorpha*, a median of seven microsatellites is required (Table 4).

**Table 4:** Microsatellite validation effort depending of the application.

Genetic application using microsatellite markers	*n*	Range	Mean (*SD*)	Median	Initial SSR markers to be validated
Genetic mapping	19	31–2000	337.89 (442.37)	209	1388.70
Individual DNA identification and parentage assignment	15	1–14	7.73 (4.03)	8	53.16
Phylogeny, population and conservation genetics	30	1–33	8.77 (6.32)	7	46.51
Molecular epidemiology and pathology	26	1–227	26.15 (49.56)	9	59.80
Quantitative trait loci mapping	13	15–428	96.54 (77.38)	77	511.63
Marker-assisted selection	6	1–7	4.17 (1.94)	4	26.58

Studies analyzed (*n*), SSR markers used and validation effort to get markers required in each genetic application using microsatellite markers. Validation effort was determined using the median values of the number of SSR used in each microsatellite-containing analysis according with 104 studies revised in Chistiakov et al. [24]. The number of locus required to be validated were calculated using the median of SSR analyzed and the polymorphism yield (15.05%) calculated in this study.

This information could be used to determine the validation effort required for a specific task. Based on the median value of SSR markers used in each genetic application with the polymorphic yield (15.05%) obtained in this study, we could estimate the number of initial SSR markers that would require validation in mollusk species depending on the desired genetic study (Table 4). Thus, for a population genetics study of eight microsatellites about 54 SSR markers must be validated. Then, the set of 14 polymorphic microsatellite markers developed on *D*. *polymorpha* clearly exceeds the median number of microsatellites required for genetic population analyses.

A preliminary analysis on the genetic structure (using a sample size of eight individuals per location) of these microsatellites in the Iberian Peninsula failed to show any population structure, but demonstrated the usefulness of these new markers. Our results suggest that all *D*. *polymorpha* individuals in the Iberian Peninsula belong to the same genetic population supporting a previous preliminary genetics study using a single microsatellite marker [51]. Since its first citation in Iberian Peninsula in the lower Ebro in 2001 [52], the expansion of *D*. *polymorpha* could be caused by a combination of a free-swimming larval dispersion and human mediated, particularly in ballast waters in vessels. Nevertheless, further analysis with a larger sample size from more locations, and even including some of the previously described microsatellites [22–24], is crucial to confirm this result.

## Conclusions

In summary, this study identified and validated 14 new polymorphic microsatellites in *D*. *polymorpha*, which, added to those developed by Naish and Gosling, Feldheim *et al*, and Thomas *et al* [22–24], increase the number of working microsatellites in this invasive species. Our results indicate that methodology based on MPS platforms and bioinformatic analysis could be used to identify a large number of markers, supporting its suitability in microsatellite isolation. This study showed that MPS approaches may be used to optimize to the development of microsatellites as genetic markers, minimizing the cost of this process.

In addition, we conducted a preliminary study of the genetic structure of *D*. *polymorpha* at six locations in Spain using all 14 new microsatellite markers. Our results showed that all six locations represent the same genetic population; however, further analysis with a larger sample size from more locations, along with more microsatellites, is crucial to confirm this result. In conclusion, this study demonstrates the usefulness of these new markers for population genetic analyses in *D*. *polymorpha*.

### Data deposition

The 454 GS FLX reads of *D*. *polymorpha* were submitted to the NCBI Sequence Read Archive under accession number SRX803533. De novo assembly was also submitted in DDBJ/EMBL/GenBank Whole Genome Shotgun project when contigs size was longer than 500 nucleotides under accession number JWHF00000000. Monomorphic microsatellite sequences were submitted in Genbank with accession numbers KP274952–KP275018, and polymorphic microsatellite sequences with accession numbers JQ812984–JQ812997.

## Supporting Information

S1 Table Samples analyzed in this study with their genetic variability statistics. (PDF)

S2 Table Number (and percentage) of microsatellite types based on the size of the repeat motif. (PDF)

S3 Table Description of the 93 validated microsatellites. (PDF)

S4 Table Allele frequencies for the 14 polymorphic microsatellite loci in the six locations. (PDF)

S5 Table Pairwise FST values among populations. (PDF)

S6 Table Number of microsatellite markers used in the different genetic analyses. (XLSX)
