# Supplementary material for: Using Massive Parallel Sequencing for the Development, Validation, and Application of Population Genetics Markers in the Invasive Bivalve Zebra Mussel (Dreissena polymorpha)
Source: PLoS One. 2015 Mar 17;10(3):e0120732. doi: 10.1371/journal.pone.0120732 (PMC4364119; doi:10.1371/journal.pone.0120732)
Supplement: S1 Table — (PDF) [file pone.0120732.s001.pdf]

S1_Table. Samples analyzed in this study with their genetic variability statistics.

n, number of samples; *A*, number of alleles (average per locus); *Ar*, allelic richness; *Hs*, gene diversity.

Study number	Study site	Basin	Coordenates		Date of collection	*n*	*A*	*Ar*	*Hs*
			Latitude, N	Longitude, E					
1	Sobron Reservoir	Ebro River basin	42° 46’	-3° 07’	2010	8	48 (3.43)	2.14	0.53
2	Canal Imperial Aragón	Ebro River basin	41° 36’	-0° 51’	2011	8	50 (3.57)	2.33	0.61
3	Ribarroja Badia-Tucana Wharf	Ebro River basin	41° 14'	0° 25'	2011	8	46 (3.29)	2.16	0.54
4	Ribarroja Reservoir	Ebro River basin	41° 14’	0° 25’	2011	8	46 (3.29)	2.29	0.60
5	Delta of Ebro River	Ebro River basin	40° 42’	0° 49’	2010	8	50 (3.57)	2.31	0.60
6	La Baells Reservoir	Llobregat River basin	42° 07’	1° 53’	2012	8	43 (3.07)	2.06	0.50
	TOTAL/AVERGE					48	67 (4.79)	2.21	0.56
