# Supplementary material for: Using Massive Parallel Sequencing for the Development, Validation, and Application of Population Genetics Markers in the Invasive Bivalve Zebra Mussel (Dreissena polymorpha)
Source: PLoS One. 2015 Mar 17;10(3):e0120732. doi: 10.1371/journal.pone.0120732 (PMC4364119; doi:10.1371/journal.pone.0120732)
Supplement: S2 Table — (PDF) [file pone.0120732.s002.pdf]

S2_Table. Number (and percentage) of microsatellite types based on the size of the repeat motif.

% of each motif Repeat Size	SSR isolation and primer design		
	SSR identified (%)	Potentially amplifically loci (%)	Loci primers designed (%)
Dinucleotide	19 (6.60)	8 (7.77)	8 (8.60)
Trinucleotide	109 (37.85)	41 (39.81)	39 (41.94)
Tetranucleotide	114 (39.58)	45 (43.69)	43 (46.24)
Pentanucleotide	46 (15.97)	9 (8.74)	4 (4.30)
Hexanucleotide	-	-	-
TOTAL	288	103	93
