# Supplementary material for: Using Massive Parallel Sequencing for the Development, Validation, and Application of Population Genetics Markers in the Invasive Bivalve Zebra Mussel (Dreissena polymorpha)
Source: PLoS One. 2015 Mar 17;10(3):e0120732. doi: 10.1371/journal.pone.0120732 (PMC4364119; doi:10.1371/journal.pone.0120732)
Supplement: S3 Table — (PDF) [file pone.0120732.s003.pdf]

S3_Table. Description of the 93 validated microsatellites.

Repeat motif, teoric size in base pairs (bp), forward (*F*) and reverse (*R*) (5'– 3') primer sequences and GenBank accession number.

Locus	Repeat motif	Teoric Size (bp)	Primers Forward (*F*) and Reverse (*R*) 5' → 3'		GenBank Accession
Dp1	$[ATA]_{21}$	262	*F:* GGATTTTTCTCCCGTGGAAT<br>*R:* CGGTAGCGTTCTCTTCACAA	Polymorphic	JQ812984
Dp2	$[TGA]_{17}$	409	*F:* GCTACCGGAGCTCAACCTAA<br>*R:* ACGTCGAACCCTGTCAAAAA	Polymorphic	JQ812985
Dp3	$[AT]_{12}$	105	*F:* TGTTAACTTGTCCATGCTATTCG<br>*R:* ATCTCAGGGGTCACAACAGG	Validated	KP274952
Dp4	$[CTA]_{59}$ 2 $[CCA]_{25}$	373	*F:* CCCATATTTACTGCCAGTGC<br>*R:* GCAGCCATTGTGTGAATACG	Not amplified	-
Dp5	$[TG]_{10}$	148	*F:* TTTCTTTTGGGAAGTCGACAC<br>*R:* ATTAGTTGCCCGGTTTCCAT	Validated	KP274953
Dp6	$[GGCT]_{11}$	256	*F:* TAACCAGGTTTTCCGAAGGA<br>*R:* GGCCATTTTTGACCTTTGAA	Validated	KP274954
Dp7	$[TAA]_{11}$ 4 $[ATT]_{5}$ 48 $[TAT]_{11}$ 71 $[ATA]_{14}$	417	*F:* GGAATACCGGGTGCTGTAGA<br>*R:* GACGTGCGTCACAATAGGTG	Polymorphic	JQ812986
Dp8	[ATC]10 [TCG]7	303	*F:* TCTATGCTCGCCAAAACGTA<br>*R:* CGGCGAAACAGTTACACAAC	Validated	KP274955
Dp9	$[CTA]_{10}$ 36 $[CTA]_{19}$	357	*F:* AATATGGGCTTCCATCCTGA<br>*R:* TGGTTATAGTGGTAGTGGTAGTGG	Validated	KP274956
Dp10	$[TGA]_{5}$ 37 $[TGA]_{10}$ 36 $[TGA]_{14}$	415	*F:* TTTGGGATATTTTGGCTGCT<br>*R:* TCCGATAAACATCGCTGTC	Validated	KP274957
Dp11	$[CAA]_{22}$ $[CAG]_{19}$ 79 $[CTA]_{8}$ 66 $[CTA]_{23}$ $[CTG]_{5}$	436	*F:* ATGACTTGTAGAAATTGCTTTGAA<br>*R:* GAACACCTTACACTTACAAATAGTCAA	Validated	KP274958
Dp12	$[ATT]_{16}$	254	*F:* CGATATACAATTGCGCCAAA<br>*R:* CAAAATAGGAATTTTGAGTTTGTGAA	Validated	KP274959
Dp13	$[CA]_{15}$	224	*F:* AACACAGGCGCATAACACAC	Validated	KP274960

			R: CATAAGCGCACTTCCGTACA		
Dp14	[ACA]$_{8}$	139	*F:* AAATTTAAATATAAACACGCATTGTG<br>*R:* AAAACAAAATCATACGCACTATTGA	Validated	KP274961
Dp15	[ATG]$_{56}$	300	*F:* TTTGTTTTTACGTGGCAGCA<br>*R:* TGAAACAAGGAAATGAGGACCTA	Not amplified	-
Dp16	[GAT]$_{10}$	212	*F:* ACAATACTCCGCTCCGAATC<br>*R:* CGACGCGCGATATTTTATTA	Validated	KP274962
Dp17	[TGCG]$_{7}$	150	*F:* TGCTCCCTCCCTTCCTTAAT<br>*R:* AAAAATGTGGTAGCCCTAGATCC	Validated	KP274963
Dp18	[GTCC]$_{13}$	104	*F:* AGAGGCGGAGGGATATTGTT<br>*R:* TATGGCTCCGGACACAAAAG	Validated	KP274964
Dp19	[AT]$_{17}$	377	*F:* TGGGATATTAATTTGGCTTCAA<br>*R:* CAATTATGGCTGACCAAGCA	Validated	KP274965
Dp20	[ATC]$_{24}$	224	*F:* CGAAAAACATATCACGCTTTCA<br>*R:* TCGTGTTTTGCTGGTTCTGA	Validated	KP274966
Dp21	[TCCG]$_{11}$	416	*F:* TTATCCCCACGCTTTTTGAA<br>*R:* GAAATTTTTGGTGGGTGTGG	Not amplified	-
Dp22	[TA]$_{23}$	246	*F:* TGCATGAGTGGTGTCAATGT<br>*R:* GTCCTCTGCACAAACCATGA	Validated	KP274967
Dp23	[TA]$_{18}$	285	*F:* TAGTCGCATGCATGAGTGGT<br>*R:* GGGCATATGTCATCCTACGG	Validated	KP274968
Dp24	[TA]$_{11}$	286	*F:* CCCAGACTGCCCATATCCT<br>*R:* CACTGGAACACCAGTCATGC	Validated	KP274969
Dp25	[CTG]$_{43}$	341	*F:* TGCAATCTTGAGAATTGTGG<br>*R:* CACAACCAGCGGTAGCATTA	Not amplified	-
Dp26	[CGGA]$_{8}$	117	*F:* TTCAATGAAATCCGCCAAAG<br>*R:* GACCTAATTATCCCCGCCATA	Validated	KP274970
Dp27	[TAG]$_{18}$	102	*F:* ATGTGCATAGTTTGGGCAGA<br>*R:* CATATTCTACTGCCAGCACCA	Validated	KP274971
Dp28	[TGA]$_{13}$	140	*F:* TAGTAGTGACGCTGCTGCTG<br>*R:* CTTTCATTCACAATTTCCACTTT	Validated	KP274972
Dp29	[CCGC]$_{9}$	119	*F:* TTTGGTGAAGATCGGATGAA	Validated	KP274973

			R: CGGGACGTATTATGTGAAACC		
Dp30	$[TTG]_7$ 58 $[GTT]_6$	218	*F:* GCGTTGGTGTTGTGTACGTC<br>*R:* CTGAGCATCTCACCGTCAAA	Polymorphic	JQ812987
Dp31	$[ATT]_{13}$	267	*F:* CGAGTTTCTTGCACGTTTCA<br>*R:* TGTTATTTTAAGAAGGCCACATTG	Polymorphic	JQ812988
Dp32	$[ATC]_{14}$ 24 $[TCG]_{12}$	348	*F:* CATCGTTATGGTCGTCATCG<br>*R:* TTTTAGCGACAAATTGACTTGG	Validated	KP274974
Dp33	$[CAT]_6$ $[TCT]_8$	447	*F:* ATCATCATCGTCGTCGTCGT<br>*R:* CACAACGGTCCGAAGTCATA	Validated	KP274975
Dp34	$[CAT]_{19}$	405	*F:* GCTAAGGGCGACAGTGTTGT<br>*R:* AACACAATGATGCTGCTGCT	Validated	KP274976
Dp35	$[GTCG]_9$	382	*F:* ACTGGACAGCAACCAGCTTT<br>*R:* TCTGACAACCACCTGAAGGA	Validated	KP274977
Dp36	$[GAAG]_{10}$	200	*F:* CTTGACCTTGACCCCATTGA<br>*R:* AATAAAATTTTGGCGGAGCA	Validated	KP274978
Dp37	$[ATG]_9$	277	*F:* TTCCCATCTTAAGTGCATTGTG<br>*R:* TTGGGTCATAGGGAGTCTGG	Validated	KP274979
Dp38	$[TCCG]_{12}$	391	*F:* TCGTCCAAATGACCAAATGT<br>*R:* GCTTTTAGTGTAGGAGGAGATAGTGG	Validated	KP274980
Dp39	$[GGCG]_{11}$	389	*F:* GACGTCATGGTTCTGAATGG<br>*R:* CCGGACAAGCTCATTTATGG	Polymorphic	JQ812989
Dp40	$[TTAA]_7$	110	*F:* GGTCAGAAGATTGGCCTCAA<br>*R:* TCGAAAGGGGGCATAAAAAT	Not amplified	-
Dp41	$[TCTG]_8$	113	*F:* AGGGGTGGGGCATTTTTAT<br>*R:* AGAGCGGACACGAAAAGTGT	Validated	KP274981
Dp42	$[GTTG]_9$	239	*F:* TCGCTTAACCTGACCAGTGA<br>*R:* CCAAATATCAAGTTGCCTATCTTCA	Polymorphic	JQ812990
Dp43	$[TTA]_{10}$	240	*F:* TTGCTCATGATGAAATATGATGT<br>*R:* ATGCGTTTCACTTTGGCATC	Polymorphic	JQ812991
Dp44	$[GACC]_8$	148	*F:* CCCCAAGCGTCTTGAGTATC<br>*R:* TCCTGCCAAGCATGTATGAG	Polymorphic	JQ812992
Dp45	$[TGT]_8$	412	*F:* GCATAACGGCTAAGTTGGTTTT	Validated	KP274982

			R: TGCTTCGTACTCAGCGATTT		
Dp46	[ACCG]$_6$	380	*F:* AGGGGTCATCTGCAAGTCAT<br>*R:* TGGGGGATATGTGTGTTGTT	Validated	KP274983
Dp47	[GAGC]$_6$	434	*F:* AGGGTGCTGCATTCTTATCG<br>*R:* GCTTGTCCAACGCAGCTATT	Not amplified	-
Dp48	[TAAA]$_5$	448	*F:* TATCCCGGCCCTATAGGAAA<br>*R:* GACAGCTCCCTCATCTTTGC	Validated	KP274984
Dp49	[AATA]$_5$	440	*F:* TGTCAATGGCGAACAGAGAA<br>*R:* GATTGGCACAAACCTGAAAAA	Validated	KP274985
Dp50	[TTA]$_6$	335	*F:* GCGCTCGATGTCAATGATTA<br>*R:* CGCGTGATTTAACAATGTCG	Validated	KP274986
Dp51	[AAAT]$_5$	301	*F:* CACAACTTTTGATTTTTGACACC<br>*R:* ATGCTGTCTGCTGGCGTTAT	Validated	KP274987
Dp52	[AAG]$_5$	208	*F:* GGCCGTTTTAAATCGTGAAA<br>*R:* TGTTTGTTTCCCCTTTCTGG	Validated	KP274988
Dp53	[TTATA]$_6$	205	*F:* TAGTGCATGCTGGGTAGCAC<br>*R:* TGGCCTTCCAGGTAAATACG	Validated	KP274989
Dp54	[ATC]$_5$	287	*F:* TGTCATCGTAAACCCAGTCG<br>*R:* AATGGGCGTAACATTTGCAT	Validated	KP274990
Dp55	[ATACA]$_6$	261	*F:* AGGGTTACACGCACAGGTTC<br>*R:* CCTACAAAACGCGCACATAC	Validated	KP274991
Dp56	[CCGT]$_5$	279	*F:* CGCAAATGTTCTATTGAGCGTA<br>*R:* CTAAGGTATGGCTCCGGACA	Validated	KP274992
Dp57	[AAC]$_5$	271	*F:* TCCCCCGTAACCAACTAACA<br>*R:* TCACTCTGGATCAGCAGACG	Not amplified	-
Dp58	[ACACC]$_7$	205	*F:* AACGACACCGCATCACTCTA<br>*R:* TGGTGTGGTGTTTATTGGAA	Validated	KP274993
Dp59	[CA]$_8$	278	*F:* AGGGTAGGGTCAACCACAACT<br>*R:* CAAAATAGTATGCGTCTCGGAAT	Not amplified	-
Dp60	[ATT]$_6$	138	*F:* CATGACACAACTTTCCCCTTA<br>*R:* CCGCTACATACCGATTGACA	Validated	KP274994
Dp61	[TCCG]$_6$	121	*F:* CGGAGGGGACTTATGGTTTT	Validated	KP274995

			R: TGCCATCTATCCATGTTCCA		
Dp62	[ACAG]$_5$	141	*F:* AGGGATGCTCCACATGAAAT<br>*R:* CTCGGGGGTGAGCTATTGT	Validated	KP274996
Dp63	[TGT]$_5$	125	*F:* TTGACGCCAACAATAACAGTTT<br>*R:* ACTGGTGGTGGGAACGATAA	Validated	KP274997
D64	[TTG]$_6$	149	*F:* TGCCAGTAGTGACGTTGGAG<br>*R:* ACAGGTCATGAACCCTCCTC	Validated	KP274998
Dp65	[TTA]$_5$	141	*F:* TCCTAACATCGATCTGTTCCAA<br>*R:* CGGCTGCTAAGGGGTAAAGT	Validated	KP274999
Dp66	[TGT]$_5$	141	*F:* GCCGTGGTTCACCTTTTT<br>*R:* AGCTAATACGCCCATTGCAT	Validated	KP275000
Dp67	[GTTG]$_7$	150	*F:* GAACATTGTTTTTGCCCTGTC<br>*R:* CCACTCAAAATGTGCAGCTC	Validated	KP275001
Dp68	[TGTTC]$_5$	287	*F:* TGCTACACACCGTATTTGCTG<br>*R:* ACACGTGGATGGTGTGAAGA	Polymorphic	JQ812993
Dp69	[GACA]$_7$	257	*F:* GGGGTCAGATCAAAATTCCA<br>*R:* TGTCCAGCATCGTCAAAAAG	Validated	KP275002
Dp70	[TTG]$_5$	212	*F:* GCAGCGACATCAGACAACAT<br>*R:* TTTCAGCTGTTTTATTGGTATGAA	Validated	KP275003
Dp71	[ACAA]$_6$	358	*F:* CTGCCATCAGCATCTGAAAA<br>*R:* TGACTGGAGTTGAGCACAGG	Validated	KP275004
Dp72	[GGTA]$_8$	383	*F:* TGCACACACATCTTGACCTG<br>*R:* GCTGAAGGCACAACATTTGA	Polymorphic	JQ812994
Dp73	[ACTG]$_5$	217	*F:* GGATGACCTTCACCTTCACC<br>*R:* ATCGCCATTGCTTTTTATGC	Validated	KP275005
Dp74	[CGTC]$_9$	361	*F:* ATCCCCTCAAGACGTTTCCT<br>*R:* ACCATACCGGTGGCATAAAA	Polymorphic	JQ812995
Dp75	[ATTT]$_6$	336	*F:* AAAAACTAGGGTAGGTAGGTACGG<br>*R:* GCACAGCATTGATGTTCTTCT	Not amplified	-
Dp76	[GACG]$_9$	312	*F:* AAATCCGCCAAAGAACTTCC<br>*R:* AAAAGGGGATTTGCCGTAAC	Validated	KP275006
Dp77	[AAAT]$_7$	259	*F:* GGAAGACCTACCTACGTTCAACC	Validated	KP275007

			R: GGTGATGGCCATGATGATTA		
Dp78	$[AACC]_6$	100	*F:* AGTTATTGCAAATGTTAAAGTTGGTG<br>*R:* CCACTAAAGTATTGGGGGACA	Validated	KP275008
Dp79	$[AAC]_5$	100	*F:* TGGTGTAAGTTAGCCATGCAA<br>*R:* TTTGTAGCTTAATTGCCGAAA	Validated	KP275009
Dp80	$[ACA]_5$	113	*F:* AGCGAACCATAGCTCGTACC<br>*R:* GCTTAAATCAAACGATTCAAGTGT	Validated	KP275010
Dp81	$[GTCT]_6$	138	*F:* TGGCAGTCTCACTGTTCACC<br>*R:* TCAAAAACGGCTAGAAAACTCC	Validated	KP275011
Dp82	$[GCAC]_6$	106	*F:* CAGAATTGGACCAGTAGTTTAGGA<br>*R:* CCACGCTAATGTCATGGTCA	Validated	KP275012
Dp83	$[ACGG]_7$	294	*F:* TGAGAAATAGCCCGGACAAA<br>*R:* GATATCCGACCTCCGTTCAA	Not amplified	-
Dp84	$[CGTC]_{11}$	364	*F:* TGTAGACCGTTTGAAACTTGC<br>*R:* AAGATCGGACAAGAAATGTGG	Validated	KP275013
Dp85	$[TCA]_{39}$	259	*F:* TTTGGGGTAAAATGCCTGAC<br>*R:* GATGATGATGGTGGTGTTGG	Validated	KP275014
Dp86	$[CGTC]_5$	312	*F:* GCAAAGGGAGAAAACTGCAC<br>*R:* CACTGTCACCGTCGCACATA	Polymorphic	JQ812996
Dp87	$[AACC]_7$	302	*F:* GATGACCTTGACCTTTCACCA<br>*R:* TGGGCATATGTCATCCTATGG	Not amplified	-
Dp88	$[TTTA]_7$	238	*F:* ATAGGCGCTCGATGTCAATG<br>*R:* CAATAAGCGAGAGGGGTGAC	Validated	KP275015
Dp89	$[CGTC]_8$	275	*F:* TTTTCACACAGCAGCCAAAG<br>*R:* TGAGAAATAGCCCGGACAAA	Polymorphic	JQ812997
Dp90	$[GGAC]_6$	216	*F:* GTTTTTGACCCAGCATGACC<br>*R:* CAAAGCGTATAGGGGGCATA	Validated	KP275016
Dp91	$[ATA]_8$	243	*F:* AAAGCGCACTTCCGTACACT<br>*R:* CGTACATGCACAACACAACG	Not amplified	-
Dp92	$[GACG]_6$	241	*F:* GAAATTGCTATCTGAAGCGACA<br>*R:* GGTCATGCAGGGTCAAAAAC	Validated	KP275017
Dp93	$[AGAC]_7$	138	*F:* TTTTGCATGATAAAAGTTCGTGA	Validated	KP275018

R: CCGGTATGTTTTCCACCACT
