# Supplementary material for: Using Massive Parallel Sequencing for the Development, Validation, and Application of Population Genetics Markers in the Invasive Bivalve Zebra Mussel (Dreissena polymorpha)
Source: PLoS One. 2015 Mar 17;10(3):e0120732. doi: 10.1371/journal.pone.0120732 (PMC4364119; doi:10.1371/journal.pone.0120732)
Supplement: S4 Table — (PDF) [file pone.0120732.s004.pdf]

S4_Table. Allele frequencies for the 14 polymorphic microsatellite loci in the six locations.

Refer to Table S1 for Sample numbers.

Locus	Allele	Ebro River Basin					Llobregat River Basin
		1	2	3	4	5	6
Dp1	299	0.1	0.2	0	0.25	0.429	0.125
	308	0.3	0.2	0.5	0.083	0.071	0.125
	314	0.2	0.2	0	0.25	0.429	0.125
	317	0.4	0.2	0.5	0.25	0.071	0.125
	323	0	0.2	0	0.167	0	0.5
Dp2	411	0	0	0	0	0.062	0
	429	0	0	0	0	0	0.1
	432	0	0	0	0.062	0.062	0
	435	0	0	0.375	0.312	0.188	0
	438	0	0	0.125	0	0	0
	441	1	0.812	0.375	0.375	0.188	0.5
	444	0	0	0.062	0.25	0.25	0.4
	453	0	0.188	0	0	0.25	0
	459	0	0	0.062	0	0	0
Dp7	430	0.438	0.625	0.125	0.688	0.375	0.214
	436	0.562	0.375	0.875	0.312	0.625	0.786
Dp30	233	0.312	0.25	0.125	0.125	0.125	0.357
	236	0.5	0.438	0.562	0.562	0.75	0.286
	242	0	0	0	0.188	0	0.286
	245	0.062	0.125	0.125	0	0	0.071
	251	0.125	0.125	0.188	0.125	0.125	0
	254	0	0.062	0	0	0	0
Dp31	250	0.125	0.062	0	0.125	0.062	0
	253	0.062	0.062	0	0.062	0.188	0.143
	262	0.125	0.062	0.312	0.375	0.25	0.214
	277	0	0	0.062	0	0	0
	280	0.062	0.125	0.125	0.188	0.062	0
	283	0.312	0.188	0.188	0.062	0.25	0.5
	295	0.062	0	0.062	0	0.125	0
	307	0.062	0.062	0	0	0	0
	310	0.062	0	0.062	0	0	0.071
	313	0.062	0.062	0	0	0	0
	325	0.062	0.312	0.125	0.188	0.062	0.071
	328	0	0.062	0	0	0	0
	337	0	0	0.062	0	0	0
Dp39	404	0.312	0.375	0.25	0.312	0.312	0.062
	408	0.188	0.125	0.312	0.375	0.188	0.75
	412	0.5	0.5	0.438	0.312	0.5	0.188
Dp42	261	0.125	0.25	0.375	0.25	0.312	0.188
	265	0.125	0.312	0.125	0.125	0.312	0.125
	277	0.75	0.438	0.5	0.625	0.375	0.688
Dp43	242	0	0	0	0	0	0.071
	260	0.812	0.625	0.75	0.938	0.688	0.857
	263	0	0	0	0	0.125	0
	269	0.062	0.125	0.188	0.062	0.188	0.071
	278	0.125	0.25	0.062	0	0	0
Dp44	156	0	0.125	0	0	0.062	0
	160	0.188	0.062	0.125	0.312	0.125	0.071
	164	0.062	0.312	0.562	0.5	0.438	0.357

	168	0	0	0	0	0.188	0
	172	0.75	0.5	0.312	0.188	0.188	0.571
Dp68	310	0.875	0.875	0.938	0.812	0.938	0.857
	315	0.125	0.125	0.062	0.125	0.062	0.143
	320	0	0	0	0.062	0	0
Dp72	397	0.562	0.25	0.25	0.438	0.188	0
	401	0.438	0.75	0.75	0.562	0.812	1
Dp74	351	0.125	0.125	0.125	0.188	0.188	0.083
	371	0.625	0.688	0.438	0.5	0.562	0.75
	379	0.25	0.188	0.438	0.312	0.25	0.167
Dp86	324	0.375	0.25	0.375	0.5	0.5	0.562
	328	0.188	0.25	0.438	0.188	0.188	0.25
	332	0.062	0.125	0.062	0.188	0.125	0.125
	336	0.375	0.375	0.125	0.125	0.188	0.062
Dp89	279	0.062	0	0	0	0.062	0
	291	0.25	0.438	0.188	0.562	0.375	0.5
	299	0.625	0.5	0.812	0.438	0.562	0.5
	303	0.062	0.062	0	0	0	0
