# Supplementary material for: Using Massive Parallel Sequencing for the Development, Validation, and Application of Population Genetics Markers in the Invasive Bivalve Zebra Mussel (Dreissena polymorpha)
Source: PLoS One. 2015 Mar 17;10(3):e0120732. doi: 10.1371/journal.pone.0120732 (PMC4364119; doi:10.1371/journal.pone.0120732)
Supplement: S5 Table — (PDF) [file pone.0120732.s005.pdf]

S5_Table. Pairwise F_{ST} values among populations.

Above diagonal: pairwise F_{ST} values. Below diagonal: P-values obtained after 300 permutations. Refer to Table S1 for sample numbers.

	Ebro River Basin					Llobregat River Basin
	1	2	3	4	5	6
1		-0.009	0.062	0.0539	0.0783	0.0985
2	0.84333		0.0451	0.0147	0.0146	0.0622
3	0.16333	0.05		0.0354	0.0276	0.0683
4	0.03667	0.1	0.26		-0.0013	0.0586
5	0.05667	0.23	0.10333	0.73667		0.0619
6	0.05	0.03	0.16	0.16333	0.15	
